# Progress towards the UNAIDS 95-95-95 targets among pregnant women in South Africa: Results from the 2017 and 2019 national Antenatal HIV Sentinel Surveys

**DOI:** 10.1371/journal.pone.0271564

**Published:** 2022-07-21

**Authors:** Selamawit Woldesenbet, Mireille Cheyip, Carl Lombard, Samuel Manda, Kassahun Ayalew, Tendesayi Kufa, Adrian Puren

**Affiliations:** 1 Center for HIV and STI, National Institute for Communicable Diseases, Johannesburg, South Africa; 2 School of Public Health, University of the Witwatersrand, Johannesburg, South Africa; 3 Centers for Disease Control and Prevention, Pretoria, South Africa; 4 Biostatistics unit, South African Medical Research Council, Cape Town, South Africa; 5 Biostatistics unit, South African Medical Research Council, Pretoria, South Africa; 6 Department of Statistics, University of Pretoria, Pretoria, South Africa; 7 Division of Virology, School of Pathology, University of the Witwatersrand, Johannesburg, South Africa; University of Ghana College of Health Sciences, GHANA

## Abstract

**Objectives:**

The UNAIDS 95-95-95 global targets for epidemic control aim to ensure by 2030 that 95% of HIV-positive people know their HIV status, 95% of people diagnosed with HIV receive sustained antiretroviral therapy (ART), and 95% of people on ART have viral suppression. While data on the first and second 95 targets are routinely reported nationally, data on the third 95 target are not available for pregnant women in South Africa. The lack of data on the third 95 target limits the inclusion of low viral suppression as one of the contributing factors in MTCT root cause analyses. This study assessed progress towards the 95-95-95 targets among pregnant women between the ages of 15–49 years attending public health facilities in South Africa.

**Method:**

Data were obtained from two consecutive national cross-sectional antenatal HIV sentinel surveys conducted between 1 October and 15 November in both 2017 and 2019. In each survey, data on age, knowledge of HIV status, ART initiation, and geographical location (province) were extracted from medical records. A blood specimen was collected from each woman and tested for HIV. Viral load tests were performed on HIV-positive specimens. Descriptive and multiple logistic regression analyses were performed to examine association between province and viral suppression (defined as viral load <50 copies/mL) using the combined dataset (i.e., both 2017 and 2019 data combined). All analyses considered the survey design.

**Results:**

Of 10 065 and 11 321 HIV-positive women included in the 2017 and 2019 surveys, respectively, 96.0% (95% confidence interval (CI): 95.6–96.4%) and 97.6% (95% CI: 97.3–97.8%) knew their HIV-positive status; 86.6% (95% CI: 85.9–87.3%) and 96.0% (95% CI: 95.6–96.4%) of those who knew their HIV status were receiving ART; while 64.2% (95% CI: 63.2–65.2%) and 66.0% (95% CI: 65.1–66.8%) of those receiving ART were virally suppressed. Achievement of the third 95 target significantly varied by province ranging from 33.9–72.6% in 2017 and 43.4–77.3% in 2019. Knowledge of HIV-positive status, ART initiation, and viral suppression increased in both 15–24 and 25–49 year age groups between 2017 and 2019. In a multivariable analysis adjusting for survey year, gravidity, and education, the odds of viral suppression significantly varied by province (except KwaZulu-Natal and Western Cape, other provinces were less likely to attain viral suppression compared to Gauteng), age (adjusted odds ratio (AOR) for 15–24 years vs 25–49 years: 0.7, 95% CI: 0.6–0.8), and timing of ART initiation (AOR for ART initiation during pregnancy vs before pregnancy: 0.4, 95% CI: 0.5–0.6).

**Conclusion:**

Although in 2019 the first and second 95 targets were achieved among pregnant women, meeting the third 95 target remains a challenge. This study highlighted the importance of promoting early ART initiation and the need to target young women in efforts to improve progress towards the third 95 target. Additionally, the provincial variation in viral suppression could be further investigated in future studies to identify and address the root causes underlying these differences.

## Introduction

In 2014, the Joint United Nations Programme on HIV/AIDS (UNAIDS) originally launched the 90-90-90 targets and recently (in 2021) updated it to 95-95-95, which aim to ensure 95% of HIV-positive people know their HIV status, 95% of people diagnosed with HIV receive sustained antiretroviral therapy (ART) and 95% of people on ART have viral suppression by 2030 [1]. The launch of these targets has galvanized tremendous efforts globally resulting in unprecedented expansion of ART access. At the end of 2019, the number of people receiving ART increased globally from just below 15 million in 2014 to 26 million (73% increase). Fourteen countries (including five Southern African countries: Namibia, Zimbabwe, Zambia, Botswana, and eSwatini) achieved the original UNAIDS targets of 90-90-90 at the end of 2019 [2].

Prevention of mother-to-child transmission of HIV (PMTCT) programs have made remarkable progress towards the earlier UNAIDS 90-90-90 targets. In 2019, 85% of HIV-positive pregnant women globally received ART, exceeding the UNAIDS first two 90 targets (*note*: *full achievement of the first two 90 targets is equivalent to achieving 81% ART coverage among all HIV-positive pregnant women*) [3]. For PMTCT, the most important target is the elimination of mother-to-child transmission of HIV (eMTCT) which aims to reduce the number of new HIV infections among children to ≤50 cases/100 000 livebirths at sub-national level in all countries, and globally to below 20 000 by 2020 [4]. For most sub-Saharan African (SSA) countries, achieving eMTCT has proven to be challenging due to the high burden of HIV among pregnant women.

South Africa is one of the high HIV burden countries in SSA with an HIV prevalence of 30% among pregnant women [5]. Despite the high HIV burden, the country has shown remarkable progress towards the UNAIDS 95-95-95 targets among pregnant women. In 2019, 97% of HIV-positive pregnant women in South Africa had received ART [3]. Through the rapid rollout of ART, MTCT rates among children in South Africa has declined from 16% (47 000 new infections) in 2010 to 3% (10 000 new infections) in 2019, with the largest decline achieved in the peripartum period [6]. Despite this progress, some gaps remain in the program that continue to challenge the achievement of eMTCT. The UNAIDS analysis of root causes of perinatal transmission of HIV in 2019 showed a large proportion of perinatal infections in South Africa occur because of two missed opportunities: pregnant women who discontinue treatment during pregnancy and women who are newly infected during pregnancy [4].

In 2019, while ART coverage among pregnant women in South Africa was reported to be 97% [3], the level of viral suppression among women receiving ART was not reported as such data were not available at the national level. The lack of viral suppression data limits the inclusion of low viral suppression as one of the contributing factors in MTCT root cause analyses. In order to fully understand missed opportunities that lead to new infant infections and inform the scale up of targeted interventions, it is useful to assess gaps in all three 95-95-95 indicators at subpopulation levels.

This study assessed progress towards the 95-95-95 targets among pregnant women aged 15–49 years attending public health facilities in South Africa using data from the 2017 and 2019 Antenatal HIV Sentinel Surveys. In an earlier analysis using the 2017 antenatal survey data, we found viral suppression among pregnant women was low (<70%) across all age groups despite the high coverage of antenatal care (ANC) testing and antenatal ART initiation; we also found ART initiation prior to pregnancy was low (55%) [7, 8]. The previous study provided baseline estimates on the UNAIDS 95-95-95 targets. We now report on progress in the achievement of the 95-95-95 targets among pregnant women by comparing the baseline estimates (for 95-95-95) reported in 2017 with data collected in the 2019 survey.

## Methods

### Study design and participants

This study used data from two consecutive national Antenatal HIV Sentinel Surveys conducted among pregnant women between the ages of 15–49 years attending ANC in public health facilities in South Africa during 2017 and 2019. The antenatal survey is a cross-sectional survey conducted every two years in all 52 districts of South Africa at sentinel sites selected using stratified cluster sampling method [5].

Each survey intended to enrol about 36 000 pregnant women from 1 595 public health facilities. This sample size allowed the detection of a 2–15% change in knowledge of HIV status, 3–7% change in ART coverage and a 4–15% change in viral suppression between two consecutive surveys at province and national level. The following assumptions were used to calculate the change in viral suppression that can be estimated between two consecutive surveys: power 80%, significance level of 0.05, proportion of sample size (n) in 2017 vs 2019 equal to 1, knowledge of HIV status (first 95) ranging between 55% to 92%, ART coverage (second 95) ranging between 90% to 96%, and viral suppression (third 95) ranging between 30% and 75% across provinces.

### Sampling and data collection procedures

Sentinel sites were selected from each of the 52 districts in South Africa using stratified (by district) probability proportional to size (PPS) cluster sampling method (using the antenatal volume of sentinel sites as a proxy for size of facility). The 2017 and 2019 surveys were conducted between 1 October and 15 November in both years. During this surveillance period, consenting pregnant women aged 15–49 years, attending the antenatal clinic for the first time or for follow-up visits during their current pregnancy, were consecutively enrolled (regardless of HIV or ART status) until either the required sample size was reached or until the end of the study period. Health workers providing ANC services in the selected facilities collected demographic and clinical data (including woman’s education, race, relationship with the father of the child, and gravidity) through interview. Data on age of the woman, current gestational age (i.e., gestational age on the day of the survey), gestational age at booking, HIV test results, timing of HIV diagnosis, and ART initiation were extracted from participants’ medical records. A blood specimen was taken from each woman regardless of prior knowledge of HIV status or ART initiation. A detailed description of the study procedures is presented in the main survey report [5].

### HIV and viral load testing

Blood specimens were tested for HIV using serial immunoassay (IA) tests: screening assay (IA-1) and confirmatory assay (IA-2). Specimens reactive on both IA-1 and IA-2 were classified as HIV-positive and tested for viral load. A detailed description of the HIV serological testing procedures (including names of test kits used) is provided in the main report [9].

HIV viral load testing was carried out on all confirmed HIV-positive (plasma) specimens using the COBAS AmpliPrep/COBAS TaqMan (CAP/CTM) HIV-1 Quantitative test (Roche Molecular Systems, Inc., Branchburg, New Jersey, USA) following the manufacturer’s manual instructions. In addition to the internal control, the assay included external controls in each run, a low positive control, high positive control, and a negative control.

### Analysis

The primary outcomes for this analysis were knowledge of HIV-positive status (first 95 target), ART initiation (second 95 target), and viral suppression (third 95 target) among women who were confirmed HIV-positive per laboratory (IA) test. Knowledge of HIV-positive status was defined as the percentage of IA positive women who knew their HIV-positive status at the time of enrolment per information extracted from medical record. ART initiation was defined as the percentage of IA positive women who knew their HIV-positive status and initiated ART (or were already on ART) per information extracted from medical record. The indicator for viral suppression used to track the third 95 target was defined as the percentage of IA positive women receiving ART whose viral load level was <50 copies/mL per laboratory viral load test done on specimens collected on the day of the survey. In addition, the percentage of women receiving ART with viral load <1 000 copies/mL is reported at national level to enable comparison with Thembisa modelling (i.e. the South African mathematical model for HIV) and other estimates. Progress towards the 95-95-95 targets were compared across demographic characteristics, province, timing of ART initiation and year of survey (i.e., between 2017 and 2019 surveys). *P* values from chi-square tests and 95% confidence intervals (CIs) were reported for such comparisons.

Data were analysed using STATA 14 (StataCorp. 2015. *Stata Statistical Software*: *Release 14*. College Station, TX: StataCorp LP) [10]. Analysis took into account the survey design (clustering within facilities and stratification by district) and was weighted for the mid-year population size of women of reproductive age (15–49 years) in the respective years at provincial level—these data were accessed from the Statistics South Africa (Stats SA) office [11, 12]. Since the sites were sampled using PPS and the sampling period was fixed, this provided a self-weighted sample at district level. A finite population correction factor was added to adjust for the >5% of facilities sampled without replacement from a finite population of about 4 000 public facilities. In the 2019 survey, the data were also weighted for sample size realization at district level as sample size achievement significantly varied by province. A multivariable logistic regression model was fitted to examine association between geographical location (province) and viral suppression. Data from both 2017 and 209 surveys were combined into one dataset for this analysis. The main variable of interest (i.e., province) and other variables significant at *P* value cut off point of 0.05 and variables that had ≥10% effect on the odds ratio of other significant variables when included in the model were kept. For the other two 95 targets (i.e for the 1^st^ 95 and 2^nd^ 95 targets), regression analysis was not done as a fairly high (>95%) achievement was observed for both targets and no geographical variation was observed.

Each analysis was done using complete observations, excluding individuals with missing values for the relevant variables. The non-response rate was ≤5% for each of the 95-95-95 indicators and for most other variables included in the current analyses in both 2017 and 2019 surveys. Participant age had >5% missing values in both surveys. A sub-sample of the missing age data was retrieved from patients’ files (in both surveys) and the retrieved data were found to have the same age distribution as the rest of age data captured from data collection forms, confirming that the exclusion of missing age data from this analysis does not introduce bias.

### Ethical considerations

Participation in the survey was voluntary, requiring written informed consent. A waiver of parental permission has been obtained from the local research ethics committee for participants in the age between 15 and 17 years. To protect the confidentiality of participants’ information, the data collection form was submitted without patient identification. HIV and viral load test results were linked with patient demographic and clinical data using unique barcodes attached to both specimen and data collection forms. Participants could withdraw from the study at any time, and this did not influence their treatment. Participants were not compensated for their participation. Ethical approval was obtained from the University of the Witwatersrand Human Research Ethics Committee (Medical) (protocol reference number: M170556) and the nine provincial health research ethics committees. The study was reviewed in accordance with the U.S. Centers for Disease Control and Prevention (CDC) human research protection procedures and determined to be research, but CDC investigators did not interact with human subjects or have access to identifiable data or specimens for research purposes.

## Results

A total of 36 128 and 41 598 participants were interviewed in the 2017 and 2019 surveys respectively; 10 358 (28.7%) participants in 2017 and 11 518 (27.7%) participants in 2019 were HIV-positive by IA test performed in the survey; 97.2% (10 065) and 98.3% (11 321) of HIV-positive participants in 2017 and 2019 surveys respectively had non-missing data for the question ‘knowledge of HIV-positive status’ and were included in the first 95 (knowledge of HIV status) analysis. Of those who knew their HIV-positive status, 98.1% (9 490) and 96.8% (10 691) had non-missing data for the question ‘participant ART status’ (based on data extracted from medical record), and 97.1% (8 087) and 94.8% (9 733) of those receiving ART had viral load data in the 2017 and 2019 surveys, respectively and these were included in the second and third 95 cascade analyses.

The socio-demographic and clinical characteristics of participants included in the current analysis are presented under S1 Table in S1 File. The demographic characteristics of participants were similar between the 2017 and 2019 surveys. The median age of participants was 28 years (interquartile range [IQR]: 24–33 years) in the 2017 survey and 29 years (IQR: 25–34 years) in the 2019 survey. Greater than 80% of participants in both surveys reported the current pregnancy was not their first pregnancy. The vast majority (97%) of participants were black African in both surveys. Just above three-quarters of participants and about 10% of participants in both surveys had attended secondary and tertiary education, respectively. Close to two-thirds of participants in both surveys were enrolled from public health care facilities in urban areas, while just below a third were enrolled from rural areas.

### Progress towards the 95-95-95 targets among pregnant women 15–49 years of age

**Fig 1:**
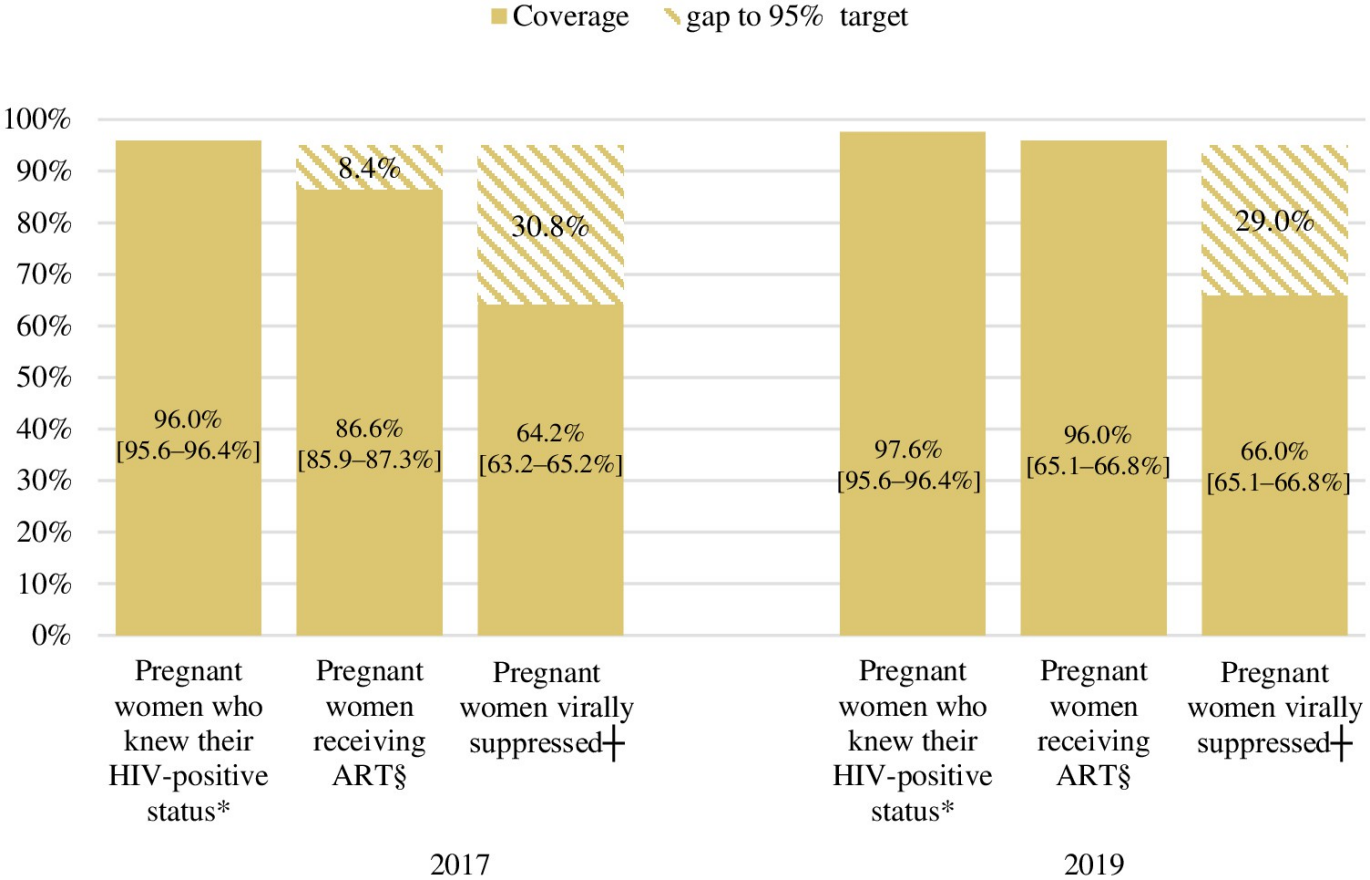
Progress towards the 95-95-95 targets among pregnant women aged 15–49 years in South Africa, in the 2017 and 2019 Antenatal HIV Sentinel Surveys. Missing data excluded from percentages calculation. Weighted percentages. ART: Antiretroviral therapy. The ranges provided in the brackets are 95% confidence intervals; Viral suppression was defined as viral load <50 copies/mL. *The denominator for knowledge of HIV status (1^st^ bars) was: the number of HIV-positive women per IA test with data on knowledge of HIV status (n = 10 065 in 2017 and 11 321 in 2019); §The denominator for receiving ART (second bars) was: the number of HIV-positive women who knew their HIV-positive status and have data on ART status (n = 9 490 in 2017 and 10 691 in 2019); and ┼The denominator for viral suppression (third bars) was: the number of HIV-positive women receiving ART and have survey viral load data (n = 8 087 in 2017 and 9 733 in 2019).

Of 10 065 and 11 321 HIV-positive women included in the 2017 and 2019 surveys, respectively, 96.0% (95% CI: 95.6–96.4%) and 97.6% (95% CI: 97.3–97.8%) knew their HIV-positive status; 86.6% (95% CI: 85.9–87.3%) and 96.0% (95% CI: 95.6–96.4%) of those who knew their HIV-positive status were receiving ART; while 64.2% (95% CI: 63.2–65.2%) and 66.0% (95% CI: 65.1–66.8%) of those receiving ART were virally suppressed (Fig 1). Of women receiving ART, 88.6% (95% CI: 88.1–89.2%) in 2017 and 86.1% (95% CI: 85.5–86.7%) in 2019 had <1 000 copies/mL viral load.

### Progress towards the 95-95-95 targets among pregnant women *by age group*

**Fig 2:**
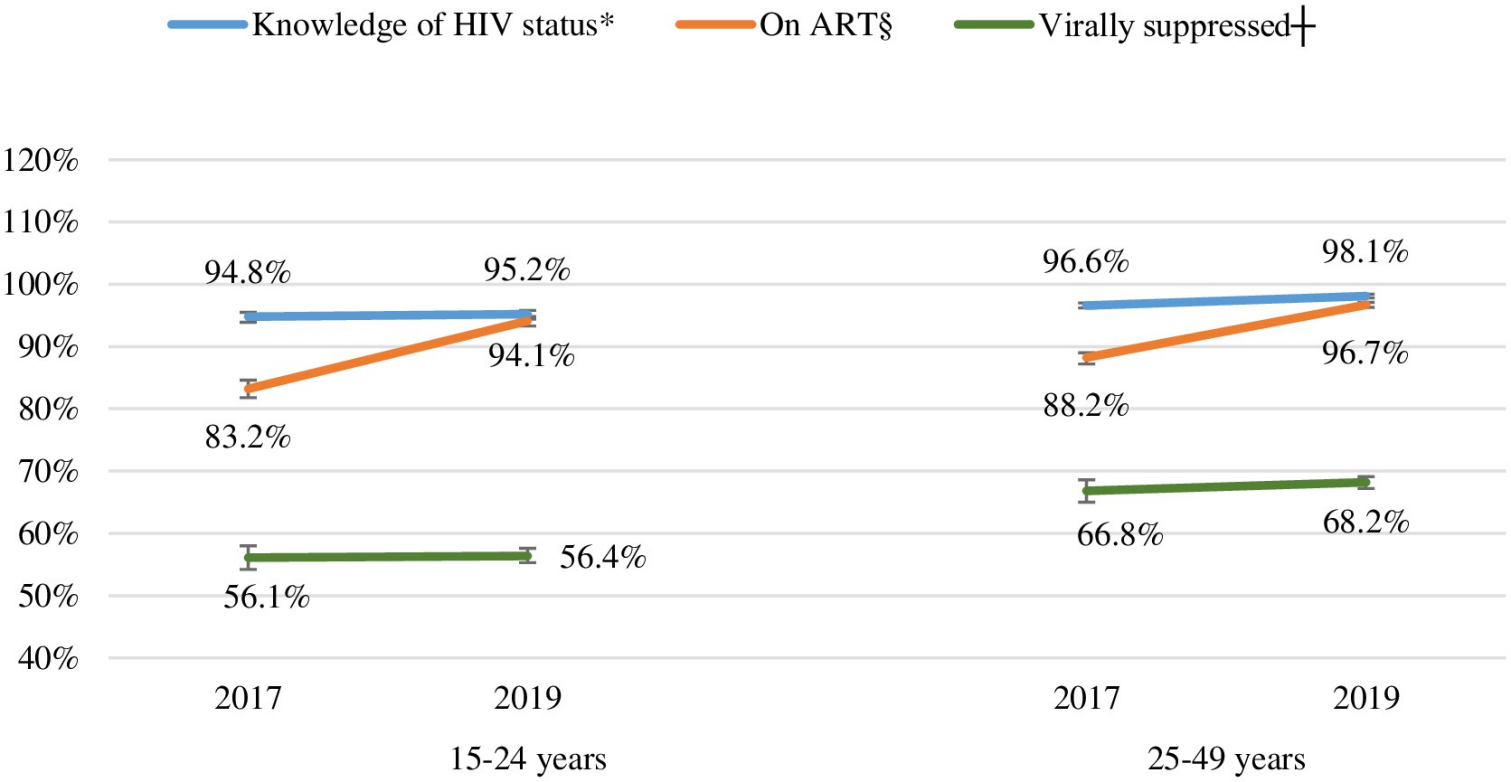
Progress towards the 95-95-95 targets among pregnant women aged 15–24 and 25–49 years of age in the 2017 and 2019 Antenatal HIV Sentinel Surveys, South Africa. Missing data excluded from percentages calculation. Weighted percentages. ART: Antiretroviral therapy. Viral suppression was defined as viral load <50 copies/mL. *The denominator for knowledge of HIV status (blue lines) was: the number of HIV-positive women with data on knowledge of HW status (n = 2 433 for women aged 15–24 years, 6 894 for 25–49 years in 2017; 2 344 for 15–24 years, 8 130 for 25–49 years in 2019). §The denominator for receiving ART (orange lines) was: the number of HW-positive women who knew their HIV-positive status and have data on ART statu (n = 2 261 for women aged 15–24 years, 6 548 for 25–49 years in 2017 and 2 160 for 15–24 years, 7 743 for 25–49 years in 2019). ┼The denominator for viral suppression (green lines) was: the number of HIV-positive women receiving ART and have survey viral load data (n = 1 865 for women aged 15–24 years, 5 667 for 25–49 years in 2017 and 1 935 for 15–24 years and 7 087 for 25–49 years in 2019). The 95% CIs for each estimates in the figure for women aged 15–24 years and 25–49 years, respectively were as follows: in 2017: for knowledge of HIV status: 93.9–95.5% and 96.2–97.0%, ART: 81.8–84.6% and 87.4–88.9%, viral suppression: 54.2–58.0% and 65.7–68.0%. In 2019: for knowledge of HIV status: 94.4–95.8% and 97.8–98.4%, ART: 93.1–94.9% and 96.3–97.1%, and viral suppression: 54.6–58.2% and 67.2–69.1%.

Knowledge of HIV-positive status, ART initiation, and viral suppression increased in both 15–24 and 25–49 year age groups between 2017 and 2019, and this increase was statistically significant for knowledge of HIV status (among the 25–49 year age group) and ART initiation (among both 15–24 and 25–49 year age groups) (*P* value <0.01 for all comparisons) (Fig 2). A lower percentage of adolescent girls and younger women (AGYW) (15–24 years) compared to older women (25–49 years) knew their HIV-positive status (94.8% vs 96.6% in 2017 and 95.2% vs 98.1% in 2019), initiated ART (83.2% vs 88.2% in 2017 and 94.1% vs 96.7% in 2019) and were virally suppressed (56.1% vs 66.8% in 2017 and 56.4% vs 68.2% in 2019) in both 2017 and 2019 surveys (*P* value <0.01 for all comparisons) (Fig 2). Progress towards the 95-95-95 targets by other demographic characteristics is presented under S2 Table in S1 File.

### Knowledge of HIV-positive status before pregnancy, ART initiation before pregnancy, and viral suppression during pregnancy among women initiated on ART before pregnancy

**Fig 3:**
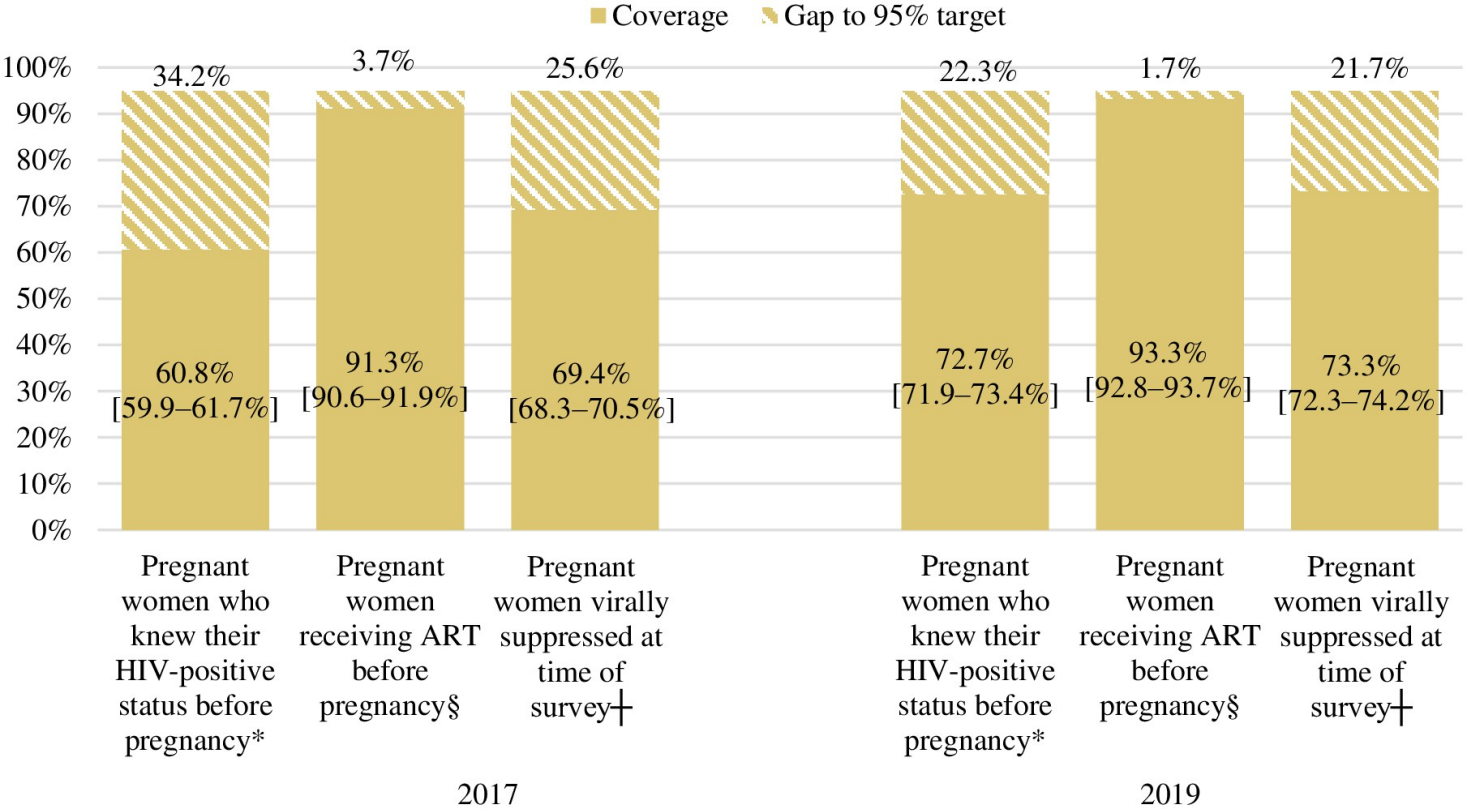
Knowledge of HIV-positive status and ART initiation before pregnancy, and viral suppression among women initiating ART before pregnancy, in the 2017 and 2019 Antenatal HIV Sentinel Surveys. Missing data excluded from percentages calculation. Weighted percentages. ART: Antiretroviral therapy. The ranges provided in the brackets are 95% confidence intervals; Viral suppression was defined as viral load <50 copies/mL. *The denominator for knowledge of HW status before pregnancy (1^st^ bars) was: the number of HIV-positive women with data on knowledge of HIV status and timing of diagnosis (n = 10 065 in 2017 and 10 778 in 2019); §The denominator for receiving ART before pregnancy was the number diagnosed before pregnancy and had data on timing of ART initiation (n = 6 167 in 2017 and 7 735 in 2019). ┼ The denominator for viral suppression (third bars) was the number on ART before pregnancy and have survey viral load data (n = 5 483 in 2017 and 6 807 in 2019).

In 2019, of the 10 778 IA positive participants with data on timing of HIV diagnosis, 72.7% (95% CI: 71.9–73.4%) were diagnosed with HIV before pregnancy—of these, 93.3% (95% CI: 92.8–93.7%) were initiated on ART before pregnancy and 73.3% (95% CI: 72.3–74.2%) of those initiated ART before pregnancy were virally suppressed at the time of the survey (Fig 3). In the same year, viral suppression among pregnant women who initiated ART before pregnancy and who were in their third trimester at the time of the survey was 74.4% (95% CI: 73.0–75.8%). Between 2017 and 2019, significant increases were observed in knowledge of HIV status before pregnancy (from 60.8% to 72.7%, *P* value <0.01), ART initiation before pregnancy (from 91.3% to 93.3%, *P* value <0.01), and viral suppression among pregnant women who initiated ART before pregnancy (from 69.4% to 73.3%, *P* value 0.01) (Fig 3). Viral suppression significantly varied by timing of ART initiation (before pregnancy: 69.4% in 2017 and 73.3% in 2019, vs during pregnancy: 52.6% in 2017 and 48.8% in 2019) (*P* value <0.01 for both comparisons).

**Fig 4:**
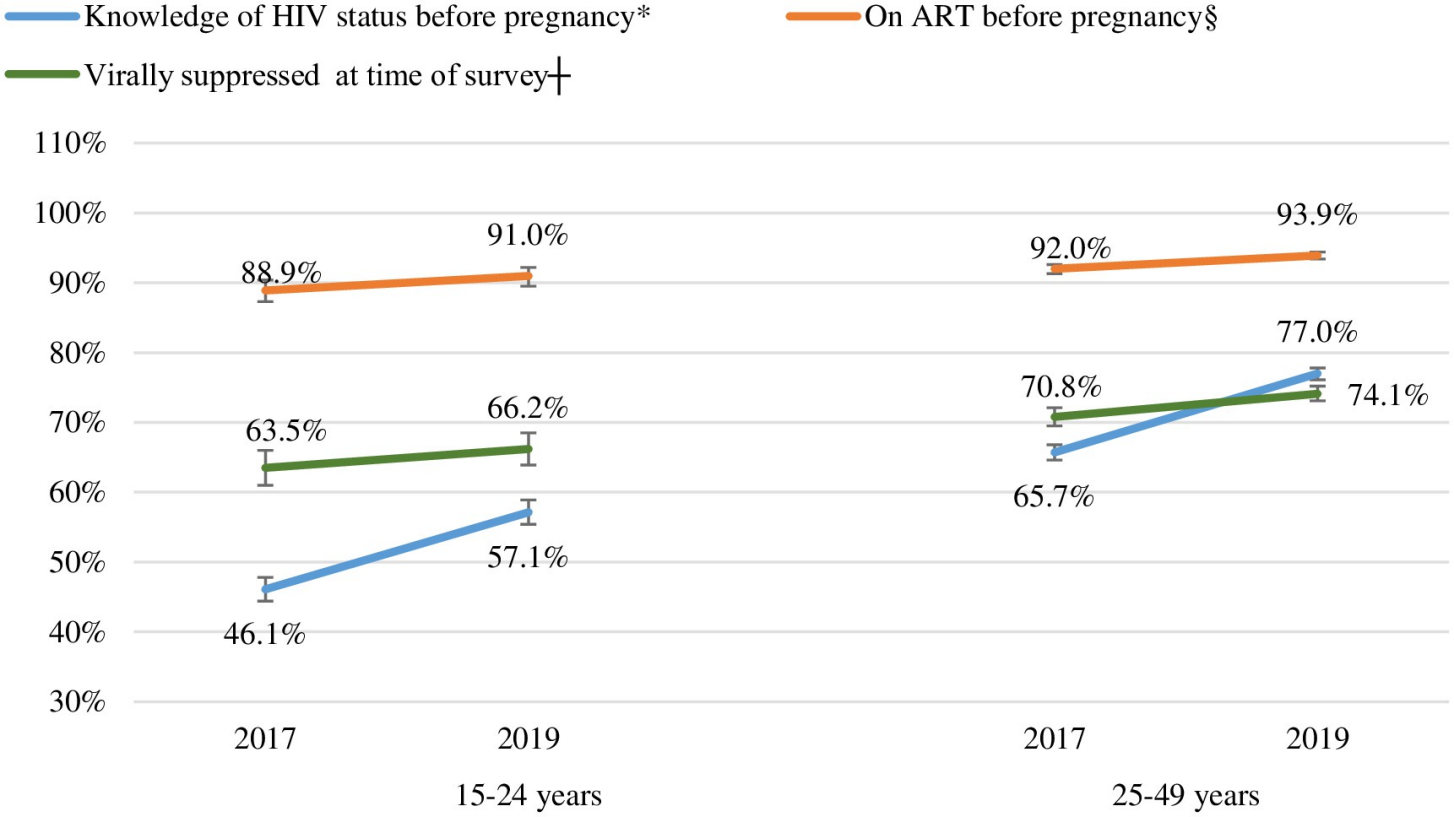
Knowledge of HIV-positive status before pregnancy, ART initiation before pregnancy, and viral suppression during pregnancy among women who initiated ART before pregnancy by age group, in the 2017 and 2019 Antenatal HIV Sentinel Surveys. Missing data excluded from percentages calculation. Weighted percentages. ART: Antiretroviral therapy. Viral suppression was defined as viral load <50 copies/mL. *The denominator for knowledge of HIV status before pregnancy (blue lines) was: the number of HIV-positive women with data on timing of HIV diagnosis (n = 2 433 for 15–24 years, 6 894 for 25–49 years in 2017; 2 209 for 15–24 years, 7 774 for 25–49 years in 2019); §the denominator for receiving ART before pregnancy (orange lines) was the number diagnosed before pregnancy and had ART data (n = 1 128 for 15–24 years, 4 590 for 25–49 years in 2017 and 1 254 for 15–24 years, 5 931 for 25–49 years in 2019); ┼the denominator for viral suppression (green lines) was the number on ART before pregnancy and have survey viral load data (n = 985 for 15–24 years, 4104 for 25–49 years in 2017 and 1 079 for 15–24 years and 5 242 for 25–49 years in 2019). The 95% CIs for each estimates in the figure for 15–24 years and 25–49 years respectively were as follows: in 2017: for knowledge of HIV status before pregnancy: 44.4–47.8% and 64.6–66.8%, ART initiation before pregnancy: 87.3–90.4% and 91.3–92.6%, viral suppression: 61.0–66.0% and 69.5–72.1%. In 2019: for knowledge of HIV status before pregnancy: 55.4–58.9% and 76.1–77.8%, ART initiation before pregnancy: 89.5–92.2% and 93.4–94.4%, and viral suppression: 63.9–68.5% and 73.1–75.2%.

Knowledge of HIV-positive status before pregnancy, ART initiation before pregnancy, and viral suppression among women initiating ART before pregnancy increased in both 15–24 and 25–49 year age groups between 2017 and 2019, and this increase was statistically significant in both age groups for knowledge of HIV status before pregnancy (*P* value <0.01 for both age groups), while for ART initiation before pregnancy and viral suppression the increase was statistically significant only among the 25–49 year age group (*P* value <0.01 for both) (Fig 4). A lower percentage of AGYW (15–24 years) compared to older women (25–49 years) knew their HIV-positive status before pregnancy (46.1% vs 65.7% in 2017 and 57.1% vs 77.0% in 2019), initiated ART before pregnancy (88.9% vs 92% in 2017 and 91.0% vs 93.9% in 2019) and were virally suppressed at the time of the survey (63.5% vs 70.8% in 2017 and 66.2% vs 74.1% in 2019) in both surveys *(P* value <0.01 for all comparisons).

### Progress towards the 95-95-95 targets among pregnant women at province level in the 2017 and 2019 surveys

Between 2017 and 2019, the number of provinces that achieved the first and the second 95 targets increased substantially. In 2017, four out of the nine provinces achieved the first 95 target and no province achieved the second and third 95 targets (S2 Table in S1 File). In 2019, seven out of the nine provinces (except Northern Cape and Western Cape) achieved the first 95 target and six of the nine provinces (except Eastern Cape, Gauteng, and Northern Cape) achieved the second 95 target. None of the provinces achieved the third 95 target in both 2017 and 2019 (Table 1). Results of the third 95 target significantly varied by province ranging from 33.9–72.6% in 2017 and 43.4–77.3% in 2019 (Table 1). Limpopo and North West had the lowest proportions of viral suppression in both 2017 and 2019 surveys. In a further analysis examining potential causes for the provincial difference in viral suppression, the difference across provinces stayed the same when viral suppression was examined by timing of ART initiation and timing of first ANC visit. There was no substantial difference between provinces in age distribution, marital status, and pregnancy intention (more details on how pregnancy intention was defined is presented in the main report) [9]. S1 Fig in S1 File presents further analysis of the 95-95-95 cascade by province.

**Table 1:** Factors associated with viral suppression among pregnant women in the 2017 and 2019 Antenatal HIV Sentinel Surveys, South Africa.

	Sample distribution n (%) N = 17 820 *	Percent virally suppressed (95% CI^┼^) 2017	Percent virally suppressed (95% CI) 2019	Unadjusted OR (95% CI)	Adjusted OR (95% CI)
**Age group (in years)**					
15–24	3 800 (22.3)	56.1 (54.2–58.0)	56.4 (54.6–58.2)	0.6 (0.6–0.7)	0.7 (0.6–0.8)
25–49	12 754 (77.7)	66.8 (65.7–68.0)	68.2 (67.2–69.1)	Ref	Ref
**Province**					
Eastern Cape	2 705 (11.6)	62.1 (59.1–65.0)	63.6 (61.6–65.4)	0.7 (0.7–0.8)	0.7 (0.6–0.8)
Free State	1 570 (4.7)	72.6 (69.8–75.1)	63.4 (60.8–66.0)	0.9 (0.8–1.0)	0.8 (0.7–0.9)
Gauteng	2 441 (25.7)	70.6 (68.6–72.5)	69.1 (67.2–70.9)	Ref	Ref
KwaZulu-Natal	5 986 (30.1)	64.1 (62.3–65.8)	77.3 (75.9–78.6)	1.0 (0.9–1.1)	1.0 (0.9–1.1)
Limpopo	839 (6.3)	33.9 (30.2–37.9)	43.4 (39.6–47.2)	0.3 (0.2–0.4)	0.2 (0.1–0.3)
Mpumalanga	1 702 (8.8)	64.1(61.4–66.7)	50.4 (47.4–53.5)	0.6 (0.5–0.7)	0.6 (0.5–0.6)
North West	1 082 (4.0)	40.0 (35.9–44.3)	47.8 (43.8–51.7)	0.4 (0.3–0.5)	0.7 (0.6–0.9)
Northern Cape	486 (2.5)	69.7 (64.2–74.7)	50.3 (44.8–55.8)	0.8 (0.6–0.9)	0.3 (0.2–0.4)
Western Cape	1 009 (6.3)	70.5 (67.5–73.3)	69.7 (66.4–72.9)	1.0 (0.9–1.1)	1.0 (0.8–1.1)
**Timing of ART** ^†^ **initiation**					
Before pregnancy	12 290 (69.8)	69.4 (68.3–70.5)	73.3 (72.3–74.2)	Ref	Ref
During pregnancy	5 145 (30.2)	53.6 (51.9–55.2)	48.8 (47.3–50.4)	0.4 (0.3–0.5)	0.5 (0.4–0.6)
**Survey year**					
2017	8 087 (48.2)	64.2 (63.2–65.2)		Ref	Ref
2019	9 733 (51.9)		66.0 (65.1–66.8)	0.4 (0.3–0.5)	1.1 (0.9–1.2)
**Education**					
None	304 (1.7)	64.2 (57.8–70.1)	64.5 (56.7–71.7)	Ref	Ref
Primary	1 974 (11.6)	61.4 (58.5–64.1)	63.1 (60.9–65.2)	0.9 (0.7–1.1)	0.9 (0.7–1.1)
Secondary	13 612 (77.0)	64.1 (63.0–65.2)	66.3 (65.3–67.3)	1.0 (0.8–1.3)	1.1 (0.9–1.4)
Tertiary	1 658 (9.8)	68.0 (65.0–70.8)	66.9 (64.5–69.2)	1.1 (0.9–1.4)	1.3 (1.0–1.6)
**Gravidity**					
Primigravida (1)	2 623 (14.5)	56.9 (54.6–59.3)	58.1 (56.0–60.2)	0.7 (0.6–0.7)	0.9 (0.9–1.0)
Multigravida (2+)	15 012 (85.5)	65.5 (64.5–66.6)	67.2 (66.3–68.1)	Ref	Ref

*Data from both 2017 and 209 surveys were combined into one dataset for this analysis.

88.7% (15 823/17 820) of observations included in multivariable analysis. Column 2 and 3 are shaded grey for year 2019 and 2017 respectively as column 2 reports viral suppression for 2017 only and column 3 reports viral suppression for 2019 only.

^┼^ CI = confidence interval;

^†^ ART = Antiretroviral therapy

### Association between geographical location (province) and viral suppression

In a multivariable analysis adjusting for survey year, gravidity, and education, the odds of being virally suppressed significantly varied by province (except for KwaZulu-Natal and Western Cape, all other provinces were less likely to attain viral suppression compared to Gauteng), age (adjusted odds ratio (AOR) for 15–24 years vs 25–49 years: 0.7, 95% CI: 0.6–0.8) and timing of ART initiation (AOR for ART initiation during pregnancy vs before pregnancy: 0.5, 95% CI:0.4–0.6) (Table 1).

## Discussion

In this national study of progress towards the 95-95-95 UNAIDS targets among pregnant women in South Africa, the first and second 95-95-95 UNAIDS targets were met in 2019 among pregnant women at national level and in five provinces, and among women 25–49 years of age. The third 95 target has not been met at either national or provincial levels. While more data are needed to assess trends in viral suppression, results from the 2017 and 2019 survey data show progress in viral suppression (third 95 target) was modest (64% to 66%). Knowledge of HIV status before pregnancy significantly improved between 2017 and 2019 across all age groups. Younger women (15–24 years) had a much lower knowledge of HIV status before pregnancy and viral suppression compared to older women (25–49 years). As can be expected, women initiating ART before pregnancy had a higher viral suppression compared to women initiating ART during pregnancy. The survey identified significant inter-provincial variation in viral suppression with the lowest viral suppression in both surveys reported in Limpopo and North West provinces.

In 2019, 310 000 livebirths to women living with HIV were reported in South Africa [4]. Applying the 95-95-95 estimates from this study on the estimated total number of livebirths to women living with HIV shows 7 440 HIV-positive pregnant women nationally in 2019 had not known their HIV status during pregnancy, 12 102 had known their HIV status but had not received ART during pregnancy, and 98 756 had received ART but had not achieved viral suppression (<50 copies/mL viral load) in 2019. The substantial number of missed opportunities (almost close to 20 000 women) for knowledge of HIV status and ART initiation, despite the achievement of the first two 95 targets, highlight that reaching the 95-95-95 targets may not be adequate to eliminate MTCT in high HIV burden countries. The implementation of services targeting the first two PMTCT prongs (prevention of HIV among women of reproductive age and prevention of unintended pregnancy among HIV-positive women) need to be strengthened to reduce infant HIV exposure and MTCT cases to a level required to achieve the eMTCT target.

Comparing 95-95-95 estimates between pregnant women in this study and women in the general population in the 2019 Thembisa model indicate progress towards the first and second 95 targets was higher among pregnant women (97.6% and 96%) in this study compared to adult females in the general population as predicted through the model (94% and 74%), which highlights the success of the PMTCT programme in meeting the first two 95 targets [13]. On the contrary, viral suppression to <1 000 copies/mL was lower among pregnant women (86%) compared to women in the general population (92% according to the Thembisa estimate). The low viral suppression among pregnant women in this study could be partially explained by the fact that some of the participants in this study (about 25%) were initiated on ART during pregnancy—most of the participants initiated on ART during pregnancy may not achieve viral suppression at the time of the survey as they may not have received ART for the minimum period of time needed for viral suppression to be achieved. Nonetheless, in this study, women who initiated ART before pregnancy also had lower viral suppression (73%) than women in the general population (92%) as predicted by the model which suggests factors other than timing of ART initiation may be responsible for the lower viral suppression among pregnant women. Lower adherence to ART during pregnancy, which may be contributed by heightened stress levels during pregnancy, fear of side effects, and suboptimal quality of adherence counselling and support in the ANC setting could explain the lower viral suppression among pregnant women [14–16]. All together these findings underscore the importance of strengthening adherence counselling and support during pregnancy as pregnant women are more vulnerable (than non-pregnant women) to individual and contextual barriers that affect adherence to treatment. Strengthening HIV testing and ART initiation before pregnancy is also critical as women initiating ART before pregnancy are most likely to have higher viral suppression during pregnancy compared to women initiating ART during pregnancy. Additionally, initiating ART during pregnancy with a more potent regimen (such as Dolutegravir) may improve faster viral suppression achievement [17].

In this study, AGYW, who represent a significant proportion of pregnant women in South Africa (about 40%), had lower knowledge of HIV status before pregnancy and lower viral suppression compared to older women (25–49 years). The lower knowledge of HIV status among AGYW may indicate gap in regular HIV testing among AGYW [18]. Late diagnosis and initiation of ART could subsequently influence achievement of viral suppression. Efforts to provide youth-friendly services and a holistic approach to reproductive health needs of AGYW need to be strengthened to promote access to HIV services, early ART initiation, and viral suppression before and during pregnancy among AGYW.

The provincial variation in viral suppression found in this survey could be further investigated in future studies to identify the root causes. In a stratified analysis, no demographic or clinical factor was found that explained the variation in viral suppression across provinces. The two provinces (Limpopo and North West) with the lowest viral suppression rate are rural provinces. The level of knowledge of MTCT could be lower in these rural areas [19]. Other factors, such as stigma and health system barriers, including poorer viral load monitoring, could be contributing factors to low viral suppression in these provinces. The he U.S. President’s Emergency Plan for AIDS Relief (PEPFAR).

South Africa provides support to high HIV burden provinces. The provinces with the lowest viral suppression in this study are provinces that have low HIV burden and low ART volume. To address the wide gap in viral suppression across provinces, support to lower volume provinces may need to be strengthened. Facility-based quality improvement initiatives have been shown to be effective in identifying and addressing gaps that contribute to poor clinical outcomes [20]. In a separate analysis of these survey data, the coverage of viral load testing and documentation of viral load test result among pregnant women in ANC sites does not significantly vary by province [21]. In South Africa, viral load test results are made available through the results for action (RFA) program—an electronic\web portal that provides easy access to viral load test results to clinicians at grassroots level. To what extent these viral load test results are used for clinical action/decision making at facility level is not known. Studies are needed to assess the utilization of these electronic viral load test results for clinical decision making. Similar to the findings in this survey, the Thembisa modelling data showed in 2019 the lowest viral suppression among reproductive age women nationally was in North West and the highest viral suppression was in KwaZulu-Natal and Free State provinces [13]. In other studies, the North West province has also been reported to have a higher (than the national average) perinatal MTCT rate [22, 23].

This survey has some limitations. The survey was restricted to public facilities, which may limit the generalizability of its findings to the overall population. The data from the two surveys in this study provided a good snapshot of the overall status/progress towards the 95-95-95 targets in South Africa. However, as data were compared only from two time points (2017 and 2019), trend analysis could not be done. More data points are needed to assess the underlying trends and distinguish true differences from differences that are due to sampling variation. Given that the pregnant women included in the two surveys are different, some of the differences in estimates observed between the two surveys could be attributable to individual variations in characteristics of women enrolled in the two surveys. Pregnant women who knew their HIV positive status before pregnancy but had not initiated treatment before pregnancy may under report their knowledge of HIV status due to social desirability bias, which may explain the low reported knowledge of HIV status before pregnancy in this study compared to estimates reported for adult females in the Thembisa model [13].

In conclusion, even though in 2019 the first and second 95 targets were met among pregnant women, meeting the third 95 target remains a challenge. Interventions to improve progress towards the third 95 target could include early ART initiation and prioritize AGYW. Viral load monitoring, use of viral load report for action, enhanced adherence counselling, peer support and family-centred interventions could be strengthened within PMTCT programmes in order to fast-track progress towards the third 95 target. Additionally, the provincial variation in viral suppression could be further investigated in future studies to identify and address the root causes underlying these differences.

## Supporting information

S1 File (DOCX)
