# Supplementary material for: Progress towards the UNAIDS 95-95-95 targets among pregnant women in South Africa: Results from the 2017 and 2019 national Antenatal HIV Sentinel Surveys
Source: PLoS One. 2022 Jul 21;17(7):e0271564. doi: 10.1371/journal.pone.0271564 (PMC9302844; doi:10.1371/journal.pone.0271564)
Supplement: S1 File — (DOCX) [file pone.0271564.s001.docx]

**Supplementary Table 1:** Socio-demographic and clinical characteristics of HIV-positive participants, in the 2017 and 2019 Antenatal HIV Sentinel Surveys in South Africa

| Description | 2017 | 2019 |
| --- | --- | --- |
|  | Number (%)  (n=10 065) | Number (%)  (n=11 321) |
| Median (IQR*) age in years | 28 (24–33) | 29 (25-34) |
| Age in years  15–19  20–24  25–29  30–34  35–49  Missing | 504 (5.0)  1 929 (20.1)  2 817 (29.8)  2 442 (26.9)  1 635 (18.2)  738 | 428 (4.2)  1 885 (17.8)  3 093 (29.3)  2 892 (27.9)  2 147 (20.8)  845 |
| Population group  Black African  Other (Coloured, white, Asian)  Missing | 9 660 (96.7)  285 (3.3)  120 | 10 824 (96.6)  452 (3.4)  45 |
| Education |  |  |
| None | 238 (2.3) | 134 (1.2) |
| Primary | 933 (9.7) | 1 406 (13.0) |
| Secondary | 7 721 (78.3) | 8 598 (75.8) |
| Tertiary | 914 (9.7) | 1 091 (10.0) |
| Missing | 259 | 92 |
| Gravidity  Primigravida (1)  Multigravida (2+)  Missing | 1 658 (16.1)  8 346 (83.9)  61 | 1 703 (15.0)  9 448 (85.0)  170 |
| Geo-type**  Urban  Rural  Peri-urban | 6 065 (64.1)  3 228 (28.7)  772 (7.2) | 6 610(62.1)  3 708 (29.8)  1 003 (8.1) |
| Province  Eastern Cape  Free State  Gauteng  KwaZulu-Natal  Limpopo  Mpumalanga  North West  Northern Cape  Western Cape | 1 330 (11.9)  877 (3.6)  1 515 (28.4)  3 325 (27.3)  575 (7.4)  1 051 (9.8)  572 (1.7)  266 (3.9)  554 (6.2) | 2 034 (12.2)  923 (5.3)  1 485 (24.1)  3 391 (28.4)  584 (7.0)  1 095 (8.6)  782 (6.4)  333 (1.4)  694 (6.7) |
| Median gestational age at the time of the survey (IQR*) | 25 (17-32) | 26 (17-32) |

*Weighted percentages; missing data not included when calculating percentages. *IQR= inter-quartile ranges. **Geo-type=geographical type*

**Supplementary Table 2:** 95-95-95 targets among pregnant women by demographic characteristic, in the 2017 and 2019 Antenatal HIV Sentinel Surveys, South Africa

| **Description** | **2017** | | | **2019** | | |
| --- | --- | --- | --- | --- | --- | --- |
|  | **First 95** | **Second 95** | **Third 95** | **First 95** | **Second 95** | **Third 95** |
|  | **% (95%CI)** | **% (95%CI)** | **% (95%CI)** | **% (95%CI)** | **% (95%CI)** | **% (95%CI)** |
| **Education** |  |  |  |  |  |  |
| None | 96.9 (94.4-98.3) | 90.8 (86.8-93.7) | 64.2 (57.8-70.1) | 98.2 (94.8-99.4) | 90.2 (83.5-94.3) | 64.5 (56.7-71.7) |
| Primary | 96.0 (94.7-96.9) | 86.5 (84.6-88.2) | 61.4 (58.5-64.1) | 98.1 (97.4-98.6) | 96.2 (95.2-97.0) | 63.1 (60.9-65.2) |
| Secondary | 96.0 (95.6-96.4) | 86.7 (85.9-87.5) | 64.1 (63.0-65.2) | 97.5 (97.2-97.8) | 96.1 (95.7-96.5) | 66.3 (65.3-67.3) |
| Tertiary | 96.2 (94.9-97.1) | 85.8 (83.7-87.6) | 68.0 (65.0-70.8) | 96.9 (96.0-97.6) | 95.6 (94.3-96.6) | 66.9 (64.5-69.2) |
| **Gravidity**  Primigravida (1)  Multigravida (2+) | 93.4 (92.2-94.4)  96.6 (96.2-96.9) | 81.6 (79.7-83.3)  87.5 (86.8-88.2) | 56.9 (54.6-59.3)  65.5 (64.5-66.6) | 95.2 (94.3-96.0)  98.0 (97.7-98.2) | 93.0 (91.8-94.1)  96.5 (96.1-96.9) | 58.1 (56.0-60.2)  67.2 (66.3-68.1) |
| **Gestational age at booking*** |  |  |  |  |  |  |
| First trimester |  |  |  | 97.8 (97.4-98.2) | 96.1 (95.5-96.6) | 69.8 (68.5-71.1) |
| Second trimester |  |  |  | 97.3 (96.9-97.7) | 96.3 (95.8-96.8) | 65.3 (64.1-66.4) |
| Third trimester |  |  |  | 97.3 (96.0-98.2) | 93.8 (91.9-95.3) | 49.0 (45.2-52.9) |
| **Gestational age at current visit** |  |  |  |  |  |  |
| First trimester | 95.6 (94.6-96.5) | 74.8 (72.7-76.7) | 67.0 (64.4-69.5) | 97.6 (96.9-98.2) | 92.7 (91.4-93.8) | 62.3 (60.2-64.3) |
| Second trimester | 95.4 (94.9-95.9) | 82.6 (81.4-83.7) | 62.0 (60.6-63.4) | 97.4 (96.9-98.1) | 95.5 (94.9-96.1) | 64.7 (63.4-66.0) |
| Third trimester | 96.9 (96.4-97.4) | 95.6 (94.9-96.2) | 65.6 (64.2-66.9) | 97.7 (97.3-98.1) | 97.8 (97.4-98.2) | 68.7 (67.4-69.9) |
| **Visit type** |  |  |  |  |  |  |
| First visit | 94.6 (93.8-95.2) | 64.8 (63.3-66.3) | 63.3 (62.2-64.4) | 97.2 (96.7-97.6) | 91.3 (90.3-92.2) | 57.0 (55.5-58.5) |
| Follow-up visit | 96.8 (96.4-97.1) | 98.2 (97.9-98.5) | 66.4 (64.7-68.2) | 97.7 (97.4-98.0) | 98.0 (97.6-98.3) | 69.4 (68.4-70.4) |
| **Geo-type****  Urban  Rural  Peri-urban | 96.4 (95.9-96.8)  95.3 (94.5-96.0)  95.9 (94.6-96.9) | 85.4 (84.4-86.4)  90.0 (88.9-91.0)  83.5 (80.8-85.8) | 66.4 (65.1-67.6)  60.0 (58.2-61.9)  62.0 (58.0-65.8) | 97.6 (97.2-97.9)  97.7 (97.2-98.1)  96.7 (95.6-97.6) | 96.4 (94.0-97.8)  97.2 (96.6-97.6)  98.5 (97.0-99.3) | 67.1 (66.0-68.3)  63.2 (61.3-65.1)  66.8 (63.8-69.6) |
| **Province** |  |  |  |  |  |  |
| Eastern Cape | 94.3 (93.1-95.3) | 84.1 (81.8-86.2) | 62.1 (59.1-65.0) | 97.2 (96.5-97.8) | 94.6 (93.6-95.5) | 63.6 (61.6-65.4) |
| Free State | 96.8 (95.7-97.6) | 88.9 (87.0-90.5) | 72.6 (69.8-75.1) | 98.3 (97.6-98.8) | 96.1 (94.6-97.2) | 63.4 (60.8-66.0) |
| Gauteng | 96.6 (95.6-97.3) | 81.6 (79.7-83.4) | 70.6 (68.6-72.5) | 97.5 (96.7-98.1) | 94.6 (93.4-95.7) | 69.1 (67.2-70.9) |
| KwaZulu-Natal | 98.3 (97.9-98.6) | 91.9 (91.0-92.7) | 64.1 (62.3-65.8) | 98.6 (98.1-98.9) | 97.5 (96.8-98.0) | 77.3 (75.9-78.6) |
| Limpopo | 93.4 (91.6-94.8) | 88.0 (85.4-90.2) | 33.9 (30.2-37.9) | 97.0 (95.5-98.0) | 96.1 (94.5-97.3) | 43.4 (39.6-47.2) |
| Mpumalanga | 93.7 (92.4-94.9) | 88.9 (86.8-90.6) | 64.1(61.4-66.7) | 97.6 (96.7-98.2) | 96.1 (94.7-97.1) | 50.4 (47.4-53.5) |
| North West | 94.9 (93.1-96.3) | 88.6 (86.1-90.7) | 40.0 (35.9-44.3) | 97.9 (97.0-98.6) | 97.2 (95.7-98.2) | 47.8 (43.8-51.7) |
| Northern Cape | 92.9(89.8-95.0) | 85.4 (81.0-88.9) | 69.7 (64.2-74.7) | 94.4 (92.1-96.0) | 94.6 (91.9-96.4) | 50.3 (44.8-55.8) |
| Western Cape | 95.5 (93.8-96.7) | 83.8 (81.4-86.0) | 70.5 (67.5-73.3) | 94.4 (92.9-95.7) | 95.7 (94.6-96.6) | 69.7 (66.4-72.9) |

**Information on gestational age at booking was not collected in 2017. Weighted percentages; missing data not included when calculating percentages. **Geo-type=geographical type*

**Supplementary section 1**

In 2019, across provinces, less than 6% of (1.8-5.8%) of women reported being unaware of their HIV-positive status at the time of the survey and between 1.5% and 5.2% of participants knew their HIV status but were not receiving ART (Supplementary Figure 1). A substantial percentage of participants (between 21.8% in Limpopo and 52.1% in KwaZulu-Natal) were initiated on ART but had not achieved viral suppression.

The denominator for each provincial bar was the total number of HIV-positive people with data for all three 95-95-95 targets which was n= 9 638 in 2017, and 10 428 in 2019 at national level

Missing data excluded from percentages calculation. Weighted percentages. ART: Antiretroviral therapy. Viral suppression was defined as viral load <50 copies/mL

**Supplementary Figure 1:** Knowledge of HIV status, treatment and viral suppression among pregnant women by province in the 2017 and 2019 Antenatal HIV Sentinel Surveys, South Africa
